# Mediterranean Diet and Multi-Ingredient-Based Interventions for the Management of Non-Alcoholic Fatty Liver Disease

**DOI:** 10.3390/nu9101052

**Published:** 2017-09-22

**Authors:** Manuel Suárez, Noemí Boqué, Josep M. del Bas, Jordi Mayneris-Perxachs, Lluís Arola, Antoni Caimari

**Affiliations:** 1 Nutrigenomics Research Group, Department of Biochemistry and Biotechnology, Universitat Rovira i Virgili (URV), Campus Sescelades, Tarragona 43007, Spain; manuel.suarez@urv.cat (M.S.); lluis.arola@urv.cat (L.A.); 2 Technological Unit of Nutrition and Health, EURECAT-Technology Centre of Catalonia, Avinguda Universitat 1, Reus 43204, Spain; noemi.boque@eurecat.org (N.B.); josep.delbas@eurecat.org (J.M.d.B.); jordi.mayneris@eurecat.org (J.M.-P.)

**Keywords:** non-alcoholic fatty liver disease, non-alcoholic steatohepatitis, hepatic steatosis, Mediterranean diet, multi-ingredient, polyunsaturated fatty acids, vitamins, synbiotics, silymarin, omics technologies

## Abstract

Non-alcoholic fatty liver disease (NAFLD) comprises a wide spectrum of hepatic disorders, from simple steatosis to hepatic necro-inflammation leading to non-alcoholic steatohepatitis (NASH). Although the prevalence of these multifactorial pathologies is continuously increasing in the population, there is still not an established methodology for their treatment other than weight loss and a change in lifestyle habits, such as a hypocaloric diet and physical exercise. In this framework, there is increasing evidence that several food bioactives and dietary patterns are effective for reversing and preventing the onset of these pathologies. Some studies have claimed that better responses are obtained when treatments are performed under a multifaceted approach, using different bioactive compounds that act against complementary targets. Thus, in this work, current strategies for treating NAFLD and NASH based on multi-ingredient-based supplements or the Mediterranean diet, a dietary pattern rich in bioactive compounds, are reviewed. Furthermore, the usefulness of omics techniques to design effective multi-ingredient nutritional interventions and to predict and monitor their response against these disorders is also discussed.

## 1. Introduction

Non-alcoholic fatty liver disease (NAFLD) comprises a wide spectrum of hepatic disorders, from simple steatosis to hepatic necro-inflammation leading to non-alcoholic steatohepatitis (NASH). Even though hepatic steatosis has been considered a benign condition for a long time, different studies have highlighted its association with cardiovascular disease (CVD), and, consequently, it is gaining relevance as a risk factor. In fact, NAFLD is defined as a metabolic liver disease in a certain metabolic context, which is associated with many metabolic diseases [1]. NAFLD is a common alteration that frequently accompanies obesity and its metabolic complications, such as insulin resistance and metabolic syndrome (MetS) [2]. Due to this link, it is estimated to affect 20% to 30% of the global population [3,4]. Despite its important prevalence, the pathogenesis of this disease remains under debate. The first hypotheses to explain its origin was known as the “two hit” hypothesis, and it considered the ectopic accumulation of fat in the liver as the trigger of second hits, which might be oxidative stress, insulin resistance or inflammation, among others [5,6]. Later, it was proposed that the progression of NAFLD was due to the hepatic lipotoxicity induced by free fatty acids, free cholesterol and different lipid metabolites, such as ceramides or lysophosphatidylcholine, and not because of the triglyceride accumulation per se. Thus, the type of the accumulated lipids, rather than the quantity, will determine the severity of this disease [5,7,8]. Nevertheless, recent evidence points to the concomitant impact of different hits as the most likely trigger of the disease [9]. Currently, a plethora of variables (including those previously listed) is considered, such as fatty acids from adipose tissue or from diet; adipocytokines, such as adiponectin or leptin; pro-inflammatory cytokines; activators of Toll-like receptors (TLR), such as lipopolysaccharide (LPS) from microbial origin; and short chain fatty acids (SCFAs) combined with endogenous causes related to the altered metabolism of lipids and lipoproteins at the gene level [10]. Therefore, despite the lack of knowledge about its pathogenesis, it is widely accepted that NAFLD can be considered a multifactorial disease.

One of the main drawbacks for the assessment and successful treatment of NAFLD and NASH is the lack of accurate non-invasive diagnosis methods. Currently, the gold standard is liver biopsy, which is recommended by the American Association for the Study of Liver Disease and the 2009 European Association for the Study of Liver Disease position statement as the endpoint for clinical trials intended to study NAFLD [11,12]. Nevertheless, liver biopsy is invasive, involves an expensive and time-consuming methodology and is not exempted from some risks to health [12]. Therefore, it is not suitable for monitoring the evolution of the disease or for general screening with the purpose of detection in early stages. Other frequently used strategies involve techniques such as ultrasonography or nuclear magnetic resonance (NMR)-based imaging, which are widely used even though they are imprecise when steatosis is in less than 30% of the hepatocytes, whereas hepatic steatosis is diagnosed for values over 5% [2,13]. Alternatively, non-invasive blood biomarkers have been widely explored [12,14]. Even though these new biomarkers are promising, research is still needed to adopt them as a general diagnostic method. Currently, the most frequently used blood biomarkers, such as hepatic enzymes alanine aminotransferase (ALT), aspartate aminotransferase (AST) or gamma-glutamyltransferase (GGT), do not offer the possibility of differentiating between the wide array of different features that usually accompany NAFLD progression, but they are accepted markers of liver damage [2].

To date, the management of NAFLD and NASH has been assessed by a plethora of clinical studies. Drug interventions have resulted in some positive outcomes, although they have been modest. Thus, insulin-sensitizing drugs, such as metformin or pioglitazone, have been successful in a limited number of cases, as have anti-hypertensive drugs, such as Losartan or Timisoara [15]. Nevertheless, drug management of NAFLD and NASH is still far from being a successful intervention, and several novel treatments based on molecules that are able to modulate key aspects of the progression of these diseases, such as farnesoid X nuclear receptor (FXR) and peroxisome proliferator activated receptor (PPAR) activities, and chemokine receptors and immune cell metabolism are under investigation [16,17,18]. 

Treatment of NAFLD and NASH remains difficult. Although bariatric surgery can be a cost-effective treatment for obese subjects with NASH regardless of fibrosis stage [19], currently, the most effective treatment for NAFLD and NASH is weight loss by a combination of dietary modifications and exercise [16,20,21,22]. Nevertheless, this approach faces different complications. Weight loss might aggravate the inflammatory status in subjects with NAFLD if it is achieved at a rate faster than 1.6 kg per week [23]. However, the most relevant handicap is that the maintenance of body weight reduction is complicated, and usually, lost weight is gradually regained [24]. This weight gain is attributed, at least in part, to the challenge of maintaining a hypocaloric diet by means of avoiding an elevated number of foods and dietary components. In this scenario, it is important to define which dietary components are prone to inducing NAFLD to design dietary interventions with higher adherence in management of the disease. Thus, even though hypocaloric diets are effective approaches to ameliorating NAFLD [21], it has been shown that the substitution of saturated fatty acids with polyunsaturated (PUFAs) or monounsaturated fatty acids (MUFAs) within isocaloric diets improves hepatic fat accumulation [25,26]. In addition, isocaloric diets with lower content of total fats have been shown to be effective in reducing liver fat content. Remarkably, the nutritional profile of the Mediterranean Diet (MedDiet), which is characterized by a high content of antioxidants and fibre, a balanced lipid profile and a low content of simple sugars, makes this diet optimal for the management of NAFLD. In fact, the beneficial effects of the MedDiet on MetS, which is closely linked to NAFLD, have been extensively evidenced [27,28]. Moreover, adherence to the MedDiet is easier than complying with the majority of the prescribed hypocaloric diets, mainly due to variety of foods and flavours, which would favour the achievement of the treatment objectives, especially in the long term. 

Another approach to the nutritional management of NAFLD consists of supplementation with specific nutrients. Thus, vitamin E supplementation resulted in positive outcomes for NASH patients in two different large clinical trials, ameliorating features such as the total NAFLD activity score (NAS) [29,30]. Despite these successful results, vitamin E therapy resulted in beneficial outcomes in 42% of subjects compared to 19% in placebo-treated patients [30], illustrating the modest success of current experimental therapies. Moreover, different meta-analyses have described the beneficial effects of omega-3 polyunsaturated fatty acids (*n-*3 PUFAs) against steatosis, although the suitability of this therapy against different features of NAFLD is not fully established [20,31]. Therefore, even though nutritional interventions are among the most successful strategies for the management of NAFLD and NASH, more research is still needed in order to identify suitable treatments with high impacts against these diseases. 

The increasing amount of evidence pointing to a multifactorial origin of NAFLD and NASH has led to strategies based on a combination of treatments in order to tackle the different hits that occur during the onset and evolution of these pathologies. The main aim of this work is to review current strategies intended to treat NAFLD and NASH by means of combining different nutritional interventions (MedDiet and multi-ingredient-based supplements), thus modulating different aspects of the disease. Furthermore, as a perspective in this field, we discuss the usefulness of omic technologies to identify the best combinations of ingredients to design effective multi-ingredient nutritional interventions and their potential for prediction and monitoring the treatment response. These strategies might be future tools for designing treatments against NAFLD and NASH.

## 2. MedDiet and Fatty Liver Management

The health benefits of the MedDiet have been widely evidenced and recognized worldwide and are mainly attributed to a lower incidence of CVD [32,33], type 2 diabetes (T2D) [34], obesity [35,36], MetS [27], some neurodegenerative diseases and certain types of cancer [37,38] and to a reduction in overall mortality [38,39]. Moreover, a number of research studies performed in the recent years have pointed to the favourable effects of the MedDiet pattern in the prevention and management of NAFLD. In this sense, an association between the MedDiet and a lower incidence or severity of NAFLD has been found in some observational studies (Table 1). Thus, in a large cohort of subjects with or without NAFLD (*n* = 532 and *n* = 667, respectively), the most powerful independent predictors of hepatic steatosis severity were body mass index (BMI), homeostasis model assessment-estimated insulin resistance (HOMA-IR) and the Adherence to Mediterranean Diet Score (AMDS) [40]. Similar results were described in two studies with smaller cohorts that used liver biopsy to diagnose NAFLD. One of them reported that adherence to the MedDiet, evaluated by the MedDiet Score, was inversely correlated with the severity of steatosis and serum ALT levels [41], whereas the other one concluded that a greater adherence to the MedDiet as estimated by the 14-Item MedDiet Assessment Tool Score was associated with a lower likelihood of having hepatic steatosis [42]. 

Furthermore, the results of several nutritional intervention studies have corroborated the beneficial effects of the MedDiet on NAFLD. Hence, in a pilot crossover study performed on 12 obese subjects with biopsy-proven NAFLD, a dietary intervention with the MedDiet for 6 weeks was able to reduce hepatic steatosis (analysed by NMR) without changes in body weight or waist circumference [43]. Fasting insulin levels and the HOMA-IR index were also decreased with the MedDiet. Interestingly, these improvements in liver steatosis and insulin resistance were not achieved after the intake of the control diet, which was a low-fat, high-carbohydrate diet based on both the Australian National Heart Foundation Diet and the American Heart Association Diet. Remarkably, no changes in serum ALT or GGT concentrations were observed after either dietary treatment. Researchers from the same group are conducting a larger parallel, multi-centre study known as MEDINA (Mediterranean Dietary Intervention study in Non-Alcoholic fatty liver disease) [50], although published results are not available at this moment. On the other hand, a recent clinical trial including 28 overweight and obese patients with ultrasonography-proven NAFLD and abnormal liver enzymes who were randomly assigned to a MedDiet or a control diet for 6 months showed that ALT levels were significantly decreased only in the MedDiet group and that AST levels tended to decrease in the MedDiet group [44]. This improvement in liver function was accompanied by greater weight loss in the MedDiet group compared to that in the control subjects. Nevertheless, liver fat content was not analysed at the end of the nutritional intervention. Gelli, et al. analysed the effectiveness of a 6-month intervention with a MedDiet to reduce the severity of NAFLD [45]. With this purpose, the authors performed a non-controlled study with 46 adults who were recently diagnosed with NAFLD. At the end of the intervention, different indices related to NAFLD and calculated through clinical and biochemical data significantly decreased: Fatty Liver Index (FLI), Kotronen index, Visceral Adipose Index and NAS. Furthermore, after the MedDiet treatment, 20% of the patients presented remission of liver steatosis, assessed by ultrasound, whereas the percentage of patients with severe steatosis decreased from 52% to 9%. This reduction of hepatic steatosis occurred along with a drop in the levels of liver enzymes ALT, AST and GGT and with a reduction in BMI and waist circumference. However, due to the lack of a control group, these findings should be carefully interpreted. 

The effects of the MedDiet supplemented or not with different bioactive compounds were evaluated in a randomized clinical trial (RCT) conducted on 30 overweight subjects with NAFLD [51]. After 6 months on a personalized hypocaloric MedDiet concomitant with physical activity recommendations, these subjects displayed significantly lower intrahepatic fat compared to baseline, as indicated by the Hamaguchi score (which evaluated the hepatic fat accumulation grade through liver ultrasound) and the FLI, even though no changes in ALT, AST or GGT concentrations were detected. This reduction of liver fat did not occur in the control group, which did not receive any treatment. In addition, body weight and waist circumference were also reduced by the MedDiet. However, it must be considered that this intervention and the one carried out by Gelli, et al. also focused on increasing the physical activity level of the participants; thus, the beneficial effects observed on NAFLD in these studies could be partially mediated by exercise. In line with these findings, another non-controlled study performed in 90 individuals with NAFLD [46] reported that an intervention based on the MedDiet and physical activity was able to reduce the severity of liver steatosis, which was evaluated by ultrasonography using the bright liver score, after 6 months, although no differences were detected following 1 or 3 months of intervention. A decrease in BMI was observed from the first month, and it stabilized between the third and sixth month of intervention. Interestingly, the authors carried out a multiple linear regression age-balanced model and found that the factors that explained the variance in fatty liver decrease in the sixth month were the changes in AMDS and BMI, independently of other factors, such as physical activity. 

The effects of a low-glycaemic MedDiet on NAFLD have been evaluated by Misciagna, et al. [47] in a RCT that included 98 men and women with moderate or severe NAFLD. The low-glycaemic MedDiet consisted of a traditional MedDiet, non-energy-restricted, including only foods with a low-glycemia index, whereas the control group followed a healthy diet recommended by the World Health Organization (WHO). After 6 months of dietary treatment, the low-glycaemic MedDiet was able to induce a greater reduction in the NAS, measured by ultrasonography, and the FLI than the control diet. The serum levels of ALT were decreased in both dietary treatments, whereas the levels of GGT were only decreased in the low-glycaemic MedDiet. 

The beneficial effects of another variant of the MedDiet, the Spanish ketogenic MedDiet, were evaluated in a pilot, non-controlled study [48] with 14 patients with MetS and NAFLD. The individuals followed a MedDiet that was restricted to a daily intake of 30 g of carbohydrates in the form of vegetables. The results of the study indicated that after 3 months of dietary treatment, these patients showed a lower degree of liver steatosis, assessed by ultrasound scans, compared to the initial value, together with a lower BMI (that dropped from 37 to 32 kg/m^2^) and waist circumference. In fact, this dietary intervention induced a complete remission of steatosis in 21.4% of the patients. Furthermore, ALT and AST serum concentrations were also decreased due to the intervention. In this regard, it seems that the failure of the MedDiet to decrease the circulating levels of hepatic transaminases observed in some of the reviewed studies could be explained by the basal levels of these enzymes in the patients who were selected for the interventions, which were already in the normal range.

After a review of all these studies, it can be concluded that the MedDiet could represent an effective dietary approach for NAFLD management, inasmuch as it reduces hepatic steatosis and improves elevated liver transaminase levels. Indeed, in the EASL-EASD-EASO Clinical Practice Guidelines for the management of NAFLD published in 2016 [52], it was recommended to adjust the dietary macronutrient composition according to the MedDiet for NAFLD treatment. However, additional large RCTs designed to identify the mechanisms underlying the observed effects are required. Furthermore, since the diets used in the above studies are fairly different in terms of macronutrient composition and food ingredients, it is also necessary to clarify the exact dietary pattern associated with the beneficial effects of the MedDiet on NAFLD.

Regarding the effects of the MedDiet on NASH, only 2 observational studies have reported an association between them. Thus, Kontogianni, et al. found that patients with NASH showed lower adherence to the MedDiet than patients with simple fatty liver and that one unit increase in the MedDietScore was associated with 36% lower likelihood of having NASH [41]. Similar results have been very recently described in pediatric patients with obesity, revealing again an association between the presence of NASH and a poorer adherence to the MedDiet [49]. To the best of our knowledge, there is no interventional study evaluating the effectiveness of the MedDiet for NASH patients.

The MedDiet is a plant-based pattern rich in grain products (preferably whole), vegetables, fruits, legumes and nuts, in addition to abundant fish intake (including oily fish), moderate consumption of eggs, poultry and dairy products (mainly yoghurt and cheese) and low consumption of red meat, processed meats and sweets [53]. Moreover, it is characterized by the use of olive oil as the main source of added fat and by a moderate consumption of alcohol during meals (mainly wine). Accordingly, the MedDiet provides plenty of nutrients and bioactive compounds that have been related to the prevention or amelioration of NAFLD, such as PUFAs [54], MUFAs [55,56], fibre [57,58], low-glycaemic index carbohydrates [59], vitamins [60,61,62,63], polyphenols [60,64], terpenes [65,66] and probiotics [67,68]. From a mechanistic point of view and as evidenced both in animal and human studies, the hepatic fat-lowering effects of PUFAs (specially *n-*3) could be mainly due to its anti-inflammatory and anti-fibrotic properties, to the up-regulation of hepatic lipolysis and fat oxidation and to the down-regulation of hepatic lipogenesis [60,69,70]. MUFAs may exert their beneficial effects on liver steatosis through the improvement of postprandial hepatic fatty acid oxidation [71,72] as well as through the stimulation of lipoprotein lipase activity in adipose tissue and the resulting enhancement of triglyceride (TAG) clearance [73,74]. In contrast, the effects of fibre intake on NAFLD could be associated with their ability to modulate the gut microbiota, leading to the subsequent attenuation of de novo fatty acid synthesis in the liver [75,76]. On the other hand, low-glycaemic index carbohydrates have been shown to induce a lower rise in postprandial glucose and insulin levels, leading to decreased activation of hepatic lipogenesis [77,78]. Regarding vitamins, those that have been demonstrated to be more effective for NAFLD patients are vitamin E and vitamin D. Specifically, different studies have reported that the mechanisms of action of these vitamins include decreased oxidative stress, lipid peroxidation, inflammation, fibrosis, lipid uptake and lipogenesis [60,63]. Likewise, the metabolic processes involved in the anti-steatotic effects of polyphenols could be the inhibition of hepatic lipogenesis, oxidative stress and inflammation and the improvement of fatty acid oxidation [60,79]. Terpenes have been found to display an anti-inflammatory effect in the liver through reduced activation of Kupffer and stellate cells as well as macrophages and T cells, which attenuates oxidative stress and the development of hepatic fibrosis and suppresses lipogenesis via the activation of AMP-activated protein kinase (AMPK) [65,80]. Finally, by shaping gut microbiota composition and decreasing endotoxaemia, probiotics have been shown to decrease liver oxidative stress, inflammation and lipogenesis [67,68,81,82]. Therefore, the beneficial effects of the MedDiet on NAFLD could be explained by both the promotion of weight loss and the supply of nutrients and bioactive compounds that may exert a direct or an indirect effect on hepatic steatosis.

Given that the MedDiet has been suggested as a natural multi-ingredient supplement [83] that may exert its related health benefits by the synergic and/or complementary action of each food compound, we will discuss the usefulness of dietary supplementation with a combination of some of these ingredients as a multi-target approach for the management of NAFLD and NASH in the next section. 

## 3. Multi-Ingredient Nutritional Approaches for the Treatment of NAFLD and NASH

In accordance with the multiple parallel-hit hypotheses, which says that the development of liver disease involves a group of alterations in the organism that occur simultaneously [84], several approaches have been performed using combinations of different bioactive compounds that act against these targets. In this framework, some studies have claimed that better results can be obtained when the treatment of NAFLD is carried out under a multifaceted approach, including different bioactive compounds that act against complementary targets, which thus avoids the progression of this disease at different levels of action [45]. Following, a review of the existing literature on clinical trials based on this approach is presented (Table 2 and Table 3).

### 3.1. Vitamin E and Silybin

Vitamin E has been described as an antioxidant agent that has a crucial role in the treatment of oxidative stress in the liver, which is one of the most important factors in the development of hepatic damage. In fact, together with weight loss, vitamin E is considered to be the only proven effective treatment against NASH in non-diabetic patients [94]. On the other hand, silybin, also known as silibinin, a flavonoid polyphenol constituent of *Silybum marianum* (the milk thistle plant), is also described as having antioxidant, anti-inflammatory and anti-fibrogenic properties. Moreover, it stimulates liver regeneration and inhibits hepatic stellate cell activation [95]. Several researchers have evaluated the combined effect of both vitamin E and silybin (along with other compounds) to identify its beneficial effect. Thus, in the study conducted by Loguercio, et al. [85], the authors evaluated the effect of a combination of silybin plus phosphatidylcholine co-formulated with vitamin E (a product known as Realsil^®^). In this RCT (multi-centre, phase III, double-blind), 138 volunteers with histologically diagnosed NAFLD (some of whom were also diagnosed as positive for Hepatitis C virus (HCV)) chronically consumed the blend of bioactive compounds twice a day during a total run-time of 12 months. The volunteers were divided into two groups, placebo and treated, and the levels of circulating liver enzymes in plasma (AST, ALT, and GGT) were improved only when the blend was consumed. In addition, the HOMA index and liver histology results were ameliorated. Regarding the BMI, it was normalized in 15% of the volunteers that consumed the blend compared to 2.1% when the placebo was consumed. Interestingly, an improvement in the fibrogenesis markers was also observed in the HCV-positive patients, but only when the blend was consumed. Although both vitamin E and silybin are described as healthy liver promoters, the authors attributed the observed beneficial effects to silybin mostly because of the low dose of vitamin E included in the tested blend (60 IU/day), which is much lower than the doses that were previously reported as effective. 

In a similar study performed some time later, Cockapoos, et al. [86] evaluated the chronic effect of a blend composed of vitamin E, an extract containing silybin and a mixture of amino acids (l-glutathione, l-cysteine and l-methionine). This blend was consumed twice daily by 72 volunteers with biometrically proven NAFLD (40 male and 32 female) for six months. Although the BMI, fasting plasma glucose, HOMA index, cholesterol and TAGs were not significantly reduced, an improvement in the levels of transaminases (ALT and AST), the Steato test, GGT, tumor necrosis factor-α (TNF-α) and the hepatorenal ratio (determined by ultrasonography) was observed in the treated group at the end of the study.

Further studies have confirmed the beneficial effect of the combination of silybin and vitamin E against NAFLD. Aller, et al. [87] went a step further and compared the effect of this blend when a lifestyle modification, including a hypocaloric diet and exercise, was performed. Thus, in their study, 36 patients diagnosed with NAFLD through biopsy were divided into two groups. One group followed the hypocaloric diet and exercised for three months while consuming the blend twice a day. The other group only followed the hypocaloric diet. Controversial results were obtained from this study. On the one hand, in both groups, anthropometric parameters, GGT, FLI and the NAFLD-Fibrosis score (FS) determined by biometric analyses decreased in a similar way. However, in the group supplemented with the blend of bioactive compounds, the levels of AST and ALT did not decrease, which is contrary to what was observed in the other group, in which the hypocaloric diet was enough to reduce these parameters. To distinguish the confounding effects produced by the weight reduction, the authors compared the parameters of the volunteers who did not reach 5% weight loss. In this case, they observed that the values of GGT, FLI and NAFLD-FS decreased only when the blend was consumed, thus confirming its effectiveness in the patients who not experience a significant reduction in body weight. Hence, the authors concluded that this type of treatment with mixtures of bioactive compounds can be an interesting alternative when other treatments and strategies have failed.

The same blend of compounds (silybin and vitamin E) and a similar approach was followed in the study conducted by Sorrentino, et al. [88], in which 78 volunteers with MetS and liver steatosis were enrolled in a study that included a hypocaloric diet and exercise for three months. Volunteers were divided into two groups, and one group was supplemented with the blend of compounds twice a day. In this case, conclusive results were obtained. Those volunteers that were supplemented with the blend of bioactive compounds showed significant improvements in most of the evaluated parameters: BMI, abdominal circumference, ultrasound liver examination, Hepatic Steatosis Index (HSI) and Lipid Accumulation Product (LAP) index. Therefore, it seems clear that the combined effect of these two compounds has a beneficial effect in the treatments aimed to reduce the damage caused by NAFLD.

### 3.2. Vitamin Mixtures

In addition to vitamin E, other vitamins have also been described as effective in the management of liver disease. Thus, vitamins D, B3, B12, A, and C have been associated with several factors that influence the pathogenesis and development of this kind of disease [63]. However, most of the studies have been performed in animal models, and there is still a need for well-designed RCTs to better understand the effects and potential applications of these bioactive compounds in nutritional therapies for the treatment of NAFLD.

An interesting study that evaluated this interaction between vitamins was performed by Kawanaka, et al. [89]; 23 volunteers with NASH orally ingested 300 mg/day of both vitamin E and vitamin C for 12 months. The effect of vitamin E against NAFLD has already been discussed. Regarding vitamin C, it has been shown to improve liver fibrosis in patients with NASH [96]. Therefore, it seemed plausible that the simultaneous ingestion of both vitamins could have a beneficial effect against fatty liver disease. Thus, although the consumption of a mixture of these two vitamins did not have any effect on the BMI of the volunteers, a significant decrease in the circulating values of ALT, thioredoxin, and high-sensitivity C-reactive protein (hs-CRP) was observed. Furthermore, analyses of liver biopsies of 10 of the volunteers showed an improvement in necro-inflammation in 80% of the cases and an enhancement of the fibrosis stage in 40% of the volunteers. In the light of these results, the authors concluded that therapies based on the combination of both vitamins could be of interest in the treatment of patients with NASH or could be used to reduce the damage produced by liver disease, delaying the progression of this type of pathology.

In another study [90], the effect of the combined consumption of vitamin C and vitamin E was compared with the results obtained with ursodeoxycholic acid treatment (an epimer of chenodeoxycholic acid that appears to replace endogenous bile acids and that has been described to have cytoprotective and immunomodulatory effects useful for the treatment of NAFLD [97]). With this aim, 57 patients with elevated levels of ALT (higher than 1.2 times the upper limit of normal) and biopsy-proven NAFLD that had followed a weight-reducing diet for three months, were enrolled in the study. Volunteers were divided in two groups. Patients in one of the groups received an oral supplement of vitamins E plus vitamin C (600 IU/day and 500 mg/day, respectively) while the volunteers in the other group received ursodeoxycholic acid (10 mg/kg/day). The intervention lasted six months, and the volunteers were evaluated every two months. Once the data were evaluated, comparable results were obtained from the two treatments. A significant decrease in AST levels was observed in both groups, and although the reduction due to the combined mixture vitamins seemed to be higher, their difference was not significant. The authors concluded that treatment with the mixture of vitamins could be a safe, low-cost and appropriate option for patients with liver disease, although more studies are needed to shed more light on this issue.

Along these lines, a RCT with a large number of participants was performed to evaluate the combined effect of a mixture of vitamin E and vitamin C plus atorvastatin (a member of the drug class known as statins) in the treatment of NAFLD [98]. Specifically, 20 mg of atorvastatin, 1 g of vitamin C, and 1000 IU of vitamin E were orally administered to 1005 patients for four years as part of the St Francis Heart Study. The results showed that, compared to the placebo, the mixture of vitamins and atorvastatin reduced the odds of developing hepatic steatosis by 71% in individuals who had NAFLD at baseline, reinforcing the idea that this vitamin combination could be a useful nutritional therapy against fatty liver disease. 

### 3.3. PUFAs and Vitamins

Another group of bioactive compounds that has been recently studied due to its potential in liver disease treatment is PUFAs, specially the group composed of *n-*3 and *n-*6 fatty acids. These bioactive compounds are widely distributed in food products, such as oils, nuts and fish. Several studies have shown that PUFAs may exert important benefits on patients with NAFLD by, for example, decreasing the fat liver content [99]. However, there is still a need for more studies on the potential use of these compounds in liver disease treatment. Therefore, several researchers are conducting studies on the effect of these compounds, both alone and in combination with others. For example, Ebrahimi-Mameghani, et al. [91] performed a study in which the combined effect of conjugated linoleic acid (CLA) and vitamin E was evaluated. In this RCT, 38 obese patients diagnosed with NAFLD were divided into two groups, and they performed the experiment for eight weeks. One group received a daily dose of three 1000 mg soft-gel capsules of CLA and 400 IU of vitamin E in addition to following a weight-loss diet. The other group received only the dose of vitamin E (400 IU) plus the weight-loss diet for eight weeks. Although several parameters, such as body weight, serum oxidative stress and lipid profile, were improved in both groups, the ALT/AST ratio, the value of fat mass and the low density lipoprotein cholesterol (LDL-C)/high density lipoprotein cholesterol (HDL-C) ratio were significantly lower in the group that received the combination of vitamin E and CLA. This fact points to a synergistic and more effective response that was produced when these two compounds were consumed simultaneously. 

Vitamin D is another micronutrient that has recently gained attention in the management of NAFLD since an association between decreased levels of vitamin D and NAFLD has been described. In fact, low levels of vitamin D correlate with the degree of steatosis. Specifically, in people with biopsy-proven NAFLD it has been shown a link between decreased levels of vitamin D and the severity of steatosis, independently of other components of the MetS [100]. In addition, some authors had described that the vitamin D receptor, which is widely expressed in the liver, plays a crucial role in the progression of fatty liver. Concretely, the expression of this receptor is negatively associated with the severity of liver histology in NASH patients [101]. Therefore, several researchers had studied the effects of vitamin D supplementation, both alone or combined with other ingredients. As an example, Amiri, et al. showed that, in NAFLD patients, the supplementation of a mixture of vitamin D plus calcium decreased ALT and fasting plasma glucose and increased HDL-C levels compared with the group that only received vitamin D [102]. Regarding the combination of vitamin D with PUFAs, Della Corte, et al. [92] performed a placebo-controlled RCT carried out with 41 children (4-16 years old) in which the effect of vitamin D plus docosahexaenoic acid (DHA) was evaluated in biopsy-proven NAFLD overweight children that had a deficiency of vitamin D. Once selected, the volunteers received a daily oral dose of vitamin D (800 IU) plus DHA (500 mg). Furthermore, the patients followed a lifestyle intervention programme that consisted of a hypocaloric diet and 1 hour of regular physical exercise twice weekly. The results showed that treatment with vitamin D plus DHA generated a reduction in the NAS, determined by a combination of liver histological parameters. Even though the FS did not change, a significant reduction in the activation of the hepatic stellate cells and in the fibrillar collagen content was observed. Furthermore, a decrease in TAGs, ALT and the HOMA index was produced by the treatment, thus confirming the healthy liver promoting activity of the selected compounds. The authors compared the results obtained in this experiment with others resulting from supplementation with only DHA [103] and significant differences were not observed among them. Therefore, the authors suggested that DHA was mainly responsible for the effects in both experiments. However, they concluded that further studies were needed in order to confirm this result.

### 3.4. Natural Extracts

Several researchers have also evaluated the effect of the ingestion of combined extracts obtained from natural sources. In general, these extracts are very rich in phenolic compounds, carotenoids, vitamins and minerals. Hence, extracts from grape seed, cocoa, pomegranate, walnuts and seeds, among others, have been tested both in vitro an in vivo in order to evaluate their potential effectiveness against NAFLD [94,104]. As an example, the study performed by Abidov et al. [93] evaluated the effect of the consumption of an extract obtained from brown marine algae (rich in fucoxanthin) together with pomegranate seed oil in 151 non-diabetic premenopausal women. The study was extended to 16 weeks and included women with and without NAFLD. At the end of the study, a significant reduction in body weight and liver fat content, determined by ultrasound scanning, was observed in both groups. However, waist circumference and the levels of plasma aminotransferase enzymes were only reduced in the NAFLD group. The authors concluded that the ingestion of both products simultaneously may have great potential in the promotion and management of obesity and liver disease.

### 3.5. Synbiotics

A growing body of scientific evidence suggests that the intestinal microbiota (IM), which represents the huge amount of microbes living in the gut, plays an important role in maintaining an optimal health status in the host through the regulation of metabolism and intestinal immune function and architecture [68,105,106]. Alterations in the composition of the gut microbiome that lead to a reduction in beneficial bacteria, also known as dysbiosis, may contribute to the pathogenesis of different disorders, such as obesity and T2D [106]. Gut-liver axis is the term used to illustrate the close interplay between the liver and the intestine, as many metabolites produced by the liver are absorbed in the intestine and around of the 70% of liver supply comes from portal vein, which carries blood from the gastrointestinal tract to this tissue [105,106]. Considering this intimate relationship between the gut and the liver, it is not surprising that dysbiosis was also associated with NAFLD and NASH [68,105,106]. Studies of faecal transplantation in mice evidenced the importance of the IM in the appearance of fatty liver, since germ-free mice that received the faecal microbiome of mice with fatty liver developed hepatic steatosis [107]. Furthermore, in humans, some studies have demonstrated that NAFLD and NASH are associated with dysbiosis, although a common pattern in the alteration of microbiome abundance was not observed among the different studies, which could be partly attributed to the differences in the obese status and the severity of the liver pathology of the individuals [105]. Thus, taking into account of all these concerns, the manipulation of intestinal bacterial communities towards a better profile emerges as a potential tool for ameliorating non-alcoholic liver disease. In this sense, the use of probiotics, which are live microorganisms—mainly *Bifidobacterium* and *Lactobacillus* bacterial strains—that confer a health benefit to the host when they are administered in adequate quantities, have appeared as a promising nutritional therapy against NAFLD [68,105,106]. Similarly, increasing evidence also suggests that prebiotics, which are selective fermented ingredients that confer health benefits to the host related to changes in the composition and/or activity of the IM, can exert beneficial effects in NAFLD [68,105,106]. These healthy effects of probiotics and prebiotics against this pathology have been recently summarized in different reviews [68,105,106] and are mainly related to an (1) improvement of dysbiosis, fatty liver severity, intestinal barrier integrity, blood lipid profile and insulin resistance; and (2) decreased hepatic lipid content and inflammation, adiposity, endotoxaemia, gut permeability and circulating levels of transaminases and pro-inflammatory cytokines. However, while most of these effects have been observed in animal models, the mechanisms by which prebiotics and probiotics exert these effects are not fully elucidated, and further RCT are required to establish the efficacy of supplementation of these bioactive ingredients against human NAFLD by evaluating hepatic lipid accumulation by liver histology [68,105,106]. 

In recent years, it has been suggested that synbiotics, a term used to describe the combined use of prebiotics and probiotics, may provide a more efficient strategy to counteract or ameliorate this pathology than the use of each bioactive alone due to the synergistic effect exerted by this combination [106]. Nevertheless, as far as we know, there are no RCTs in which a comparison between the effects of the separate and combined administration of prebiotics and probiotics against NAFLD or NASH have been evaluated. This section will be focused on reviewing the data obtained in different RCTs in which symbiotic supplementation was used as a therapy against non-alcoholic liver disease (Table 3). 

In a well conducted placebo-controlled study that included 52 adult overweight and obese patients with NAFLD, Eslamparast and co-workers analysed the effects of the supplementation with fructooligosaccharide (FOS) plus a mix of seven bacterial strains (*L. casei*, *L. rhamnosus*, *L. acidophilus*, *L. bulgaricus*, *Streptococcus thermophilus*, *B. breve* and *B. longum*) twice daily in combination with the adherence to an energy-balanced diet and physical exercise for 28 weeks [108]. The authors reported that this multi-ingredient produced a significantly greater decrease in the FS (analysed by transient elastography) and the circulating levels of the transaminases ALT, AST and GGT than the administration of the placebo combined with lifestyle modification [108]. In addition, the subjects that received the synbiotic also displayed a significant drop in the circulating levels of pro-inflammatory cytokines TNFα and CRP and an improvement in insulin resistance as evidenced by decreased blood glucose and HOMA-IR [108]. Some of these effects were already clearly observed 14 weeks after the beginning of the treatment [108]. Interestingly, in a very similar RCT, the same group recently demonstrated the usefulness of this nutritional strategy in the management of NAFLD in subjects with a normal or low BMI [109]. They observed a greater reduction of hepatic steatosis and fibrosis, the circulating levels of glucose, TAGs, AST and CRP in these patients than in the participants allocated to the placebo group [109]. Furthermore, in both studies, the authors reported a significant decrease in the p65 subunit of the master regulator of inflammation nuclear factor k-B (NF-kB p65) in peripheral blood mononuclear cells (PBMCs) [108,109], suggesting that the synbiotic could partly exert its anti-inflammatory effects by inhibiting the activation of this protein. In addition, in obese and overweight subjects, the decreased activity of NF-κB p65 may account for the reduction in TNFα secretion and the consequent attenuation of insulin resistance [108]. They also hypothesized that the improvement of dysbiosis and intestinal permeability could also play a role in the beneficial effects of this multi-ingredient, since the translocation of toxic bacterial products, such as lipopolysaccharides (LPS), from the intestine to the circulation contributes to the pathogenesis of NAFLD through the enhancement of oxidative stress and inflammation, insulin resistance and hepatic fat accumulation [108,109]. In addition, the increase in the bacterial production of SCFAs, which stimulates hepatic lipid oxidation, modulates insulin sensitivity and exerts anti-inflammatory and antioxidant effects in the intestine, could also account for the observed effects [109]. Nevertheless, the authors neither analysed the composition of the IM, blood endotoxaemia and SCFAs nor evaluated NAFLD by means of a liver biopsy before and after supplementation of the synbiotic [108,109]. Therefore, despite these very promising and consistent results, further studies are needed to confirm the effectiveness of the combined treatment against NAFLD and to shed more light on the mechanisms involved in the observed effects. 

Intriguingly, the effects of this symbiotic on NAFLD have also been recently evaluated in three additional pilot RCTs with conflicting results [110,111,112]. Thus, in a study carried out with 74 overweight and obese volunteers who did not undergo dietary and physical exercise programmes, one capsule per day of this multi-ingredient significantly decreased the severity of hepatic steatosis (analysed by ultrasonography) 8 weeks after the beginning of the treatment, fully counteracting this alteration in 50% and 25% of the participants who showed a mild and moderate degree of NAFLD at baseline, respectively [110]. Although this attenuation of fatty liver was only weakly observed in the group that received the placebo, when a direct comparison between both groups was performed, no significant changes were reported in this parameter at the end of the treatment period, and the synbiotic did not produce a significant decrease in the circulating levels of ALT, AST or CRP [110]. In another study conducted with 60 adult overweight and obese participants with NAFLD diagnosed by hepatic ultrasonography and elevated levels of ALT, Ekhlasi and co-workers analysed whether daily supplementation with this multi-ingredient (two capsules per day) plus vitamin E (400 IU/day) for 8 weeks strengthened the effects produced by the synbiotic or the vitamin E when they were administered separately [111,112]. Although the volunteers were not subjected to a lifestyle modification programme, the authors found that both the synbiotic and the symbiotic plus vitamin E produced a similar decrease in systolic blood pressure (SBP) and the circulating levels of TAGs and total cholesterol [111,112]. However, in comparison to the placebo group, the combined administration of symbiotic and vitamin E produced a greater drop in the circulating levels of LDL-C, TNFα, ALT, AST, ALP, leptin, insulin and glucose than supplementation of the symbiotic alone [111,112]. These results suggested that the synbiotic plus vitamin E was generally the most effective treatment in lowering parameters closely related to the pathogenesis of NAFLD [111,112]. Nevertheless, the authors did not analyse the effects of the different treatments on hepatic steatosis by ultrasonography or liver biopsy; therefore, further RCTs are needed to elucidate whether the supplementation of the symbiotic plus vitamin E is more effective that treatment with the symbiotic alone against NAFLD [111,112]. 

The discrepancies among the different studies that have evaluated the effectiveness of this synbiotic against NAFLD may rely on the length of the treatment, the daily dose, the inclusion or absence of a lifestyle modification programme, the inclusion criteria, the sample size and even the statistical approach used to analyse the data (Table 3) [108,109,110,111,112]. Considering all the data, supplementation with this multi-ingredient twice daily in combination with a dietary treatment and moderate physical training for 28 weeks could be a useful strategy to consistently ameliorate hepatic steatosis, insulin resistance and the circulating markers of hepatic damage and inflammation that accompany NAFLD. However, additional RCTs that include liver biopsy results and are carried out with a larger number of participants are crucial to confirming the effectiveness of this nutritional therapy. 

Two additional RCTs have also evaluated the ability of synbiotics combined with a lifestyle modification to ameliorate NASH in biopsy-diagnosed subjects [113,114]. In a study performed with 66 adult patients, supplementation with FOS plus *B. longum* twice daily combined with an energy-balanced diet and physical training for 24 weeks produced a greater reduction in the NASH activity index, which was evaluated by liver histology and included the analyses of steatosis, parenchymal inflammation, and hepatocellular injury, than lifestyle modification alone [113]. Interestingly, 67% of patients supplemented with this synbiotic showed a histological response, and on average, the index decreased from 9.44 at baseline to 3.22 at the end of the study [113]. Furthermore, the subjects that received the synbiotic also presented a more pronounced decrease in the surrogate marker of insulin resistance HOMA-IR and the circulating levels of AST, LDL-C, TNFα and CRP than the placebo group, with the differences between both groups reaching statistical significance [113]. The authors partly attributed these beneficial effects to the greater decrease in serum endotoxin levels observed in the synbiotic group, which in turn would ameliorate oxidative stress and the hepatic and systemic inflammatory status through the inhibition of TNFα secretion from hepatic macrophages [113]. In another RCT, Ferolla et al. evaluated the effects of the administration of 4 grams of inulin and partially hydrolysed guar gum plus L. reuteri twice daily for 3 months in 27 adult subjects with NASH and MetS that were submitted to a hypocaloric diet (1800 Kcal/day for men and 1500 Kcal/day for women) [114]. When compared to the values obtained at baseline, supplementation with the synbiotic produced a significant decrease in liver steatosis, which was measured by proton density fat fraction (PDFF), and this effect was not observed in the 23 individuals who received the placebo and dietary treatment [114]. Despite the interest of this finding, it is important to remark that at baseline, even though the grades of steatosis identified in the liver biopsies before inclusion were similar between both groups, the median values of PDFF were significantly higher in the synbiotic group than in the placebo-treated patients (14.9 versus 6.4, *p* = 0.040). In addition, at the beginning of nutritional treatment, the number of subjects with a moderate or severe degree of steatosis (grade 2–3) was numerically higher in the multi-ingredient-supplemented group than in its counterpart (11 versus 4, *p* = 0.073) [114]. Unlike what was observed in the placebo group, the individuals who received the synbiotic also displayed a slight, but significant, drop in BMI, waist circumference and body weight at the end of the study compared to the values obtained at baseline [114]. Another remarkable positive finding of this RCT was the reduction in the circulating levels of uric acid found in the subjects supplemented with the synbiotic [114]. Uric acid is a risk factor for NAFLD [114], and it could be involved in the development of MetS and diabetes [115], pathologies that are often associated with this hepatic pathology. Nevertheless, the symbiotic did not improve either liver fibrosis–determined by elastography–or the circulating levels of glucose, lipids, ALT, AST, ALP and GGT [114]. Intriguingly, both groups of subjects displayed increased circulating levels of LPS at the end of the study, suggesting that a decrease in blood endotoxaemia would not account for the beneficial effects of the symbiotic against NASH [114], unlike what was hypothesized by Malaguarnera et al. [113]. Despite both studies showing promising results for synbiotic therapy combined with lifestyle modification against NASH, the RCT carried out by Malaguarnera and co-workers [113] revealed more conclusive results than those conducted by Ferolla et al. [114], suggesting that supplementation with FOS plus *B. longum* is more effective than treatment with inulin, guar gum and L. reuteri. The higher effectiveness of the synbiotic used by Malaguarnera et al. could also rely on the longer period of treatment, the slightly larger sample size, the implementation of a mild physical training plan and the realization, at baseline and at the end of the study, of a liver biopsy, which is the gold standard for NAFLD/NASH and provides a more accurate measurement of steatosis than Magnetic Resonance Imaging (MRI) techniques. 

Summarizing, the data obtained to date suggest that synbiotics exert protective effects against NAFLD and NASH, improving liver steatosis and/or fibrosis and some metabolic parameters closely related to these pathologies when they are combined with a lifestyle modification. However, there is a need to carry out more RCTs in which liver biopsies are performed at the end of treatment, involving a larger number of participants and addressing the elucidation of mechanisms involved in the observed effects. In this sense, analyses of gut microbiome composition, blood endotoxaemia and faecal and circulating levels of SCFAs are needed to provide a deeper understanding of the mechanisms by which synbiotics can exert healthy effects against these liver pathologies. Additional measurements, such as glucose tolerance tests, cholesterol and bile acid faecal levels and the expression of genes and proteins involved in immune function and inflammation, lipid and glucose metabolism and insulin signalling in the liver and/or PBMCs, could also be very useful in shedding more light on the mechanisms underlying the beneficial effects of these multi-ingredients against the alterations that accompany NAFLD and NASH, such as inflammation, insulin resistance and dyslipidaemia.

## 4. Application of Omics Technologies in Liver Diseases

### 4.1. Role of Omics Technologies in Evaluating the Pathogenesis of NAFLD and NASH

The mechanisms underlying the aetiology and progression of NAFLD are far from being fully understood. In addition, susceptibility to NALFD in the determination of the progressive phenotype is highly variable. Thus, it remains unclear why only some individuals who are obese develop steatosis and why only a fraction of at-risk individuals develop NASH. Because of all this, no treatment has shown clear efficacy in the prevention of NAFLD and many of the nutritional interventions against NAFLD only showed success in a limited percentage of participants [116,117]. However, the recent developments in non-hypothesis-driven, high-throughput omics technologies, such as genomics, epigenomics, transcriptomics, proteomics and metabolomics, which allow the study of complete profiles of genes, gene expression, proteins or metabolites, have shed some light on the pathogenesis of NAFLD [118]. 

Genetic predisposition can be assessed by genome-wide associations studies (GWAS). In contrast to candidate-gene studies, GWAS are particularly useful for the identification of novel genes associated with diseases that otherwise would have not been considered as candidates due to a limited knowledge of their function. To date, there is compelling evidence of the association of variants in the Patatin-like phospholipase domain containing 3 (PNPLA3) and transmembrane 6 superfamily member 2 (TM6SF2) genes, which are both involved in liver fat retention through lipid droplet remodelling and very-low density lipoprotein (VLDL) secretion, and NAFLD susceptibility and progression to NASH [119,120,121,122,123]. On the other hand, environmental factors and their impact on pathways involved in the pathogenesis of disease can be assessed by other omics analysis. Thus, using transcriptomic, proteomic, and metabolomic technologies, genome-wide changes can be assessed at the RNA, protein, and metabolite level, respectively. Global mRNA transcript expression profiling by microarray has been performed in humans with NAFLD. A recent meta-analysis of transcriptomic datasets from liver biopsies revealed a common gene expression signature of 22 genes related to lipogenic and cholesterol metabolic pathways that correlated with the progression of disease from steatosis to NASH [124]. Another microarray analysis aimed at characterizing gene expression in percutaneous liver biopsies identified a 20-gene profile that independently correlated with the severity of liver fibrosis [125]. While transcriptomics allows the identification of alterations in gene expression, proteomics can be used to monitor changes in protein expression and post-translational modifications at different stages of disease progression. Proteomic studies of NAFLD have revealed 34 candidate biomarkers in liver and serum [119,122,126]. Gene or protein expression changes do not always correspond to phenotypic alterations because they operate on a markedly different time scales. In contrast, metabolic profiles provide a snapshot of the biochemical state of an individual at a particular time. The metabolic profile is determined by both host genetic (endogenous metabolites) and environmental factors (exogenous metabolites), and it reflects the processes that have taken place or are ongoing [127]. Consequently, they monitor the global outcome of all the influencing factors and have the strongest correlation with the phenotype. As such, metabolomics provides a powerful tool for understanding complex diseases such as NAFLD [128]. Recently, there has been growing evidence of the contribution of epigenetic alterations and the IM in the development and progression of NAFLD. Therefore, new omics layers, such as epigenomics [129,130,131] and metagenomics [105,132], may also potentially contribute to our understating of the pathogenesis of this condition. 

Although studies of a single omics data type may provide valuable information for markers of disease and related biological pathways, they only provide information at a single layer of biological regulation; thus, the results are only associative. In contrast, complex diseases result in phenotypes that are likely to result from a complex interplay at multiple levels of biological regulation. Thus, a thorough and comprehensive understanding of the mechanisms or causative effects of a complex disease like NAFLD can only be achieved by integrating multiple omics technologies to form a complete picture of the complete biological system [133,134]. Thus, in order to obtain a more comprehensive view of NAFLD, several multi-omic approaches have recently been applied [124,135]. 

### 4.2. Omics Technologies for Nutritional Interventions against NAFLD and NASH

So far, only a limited number of studies have applied omics technologies to design multi-ingredients against NAFLD and NASH. Considering the potential of these techniques to provide a better understanding of the molecular mechanisms underlying the pathogenesis of NAFLD and that the recent technological advances have made these omics platforms more cost-effective, they may also provide valuable information for the identification of targets and the discovery of biomarkers that may be used to design effective combinations of ingredients for NAFLD treatment and prevention. 

In a recent study, Mardinoglu et al. applied a genome-scale metabolic model (GEM) and untargeted plasma metabolomics to gain more insight into the molecular mechanisms leading to the occurrence of hepatic steatosis [136]. Eighty-six subjects with varying degrees of hepatic steatosis were classified into two groups (low or high hepatic steatosis) based on their liver fat percentage. The secretion rates of VLDLs from the livers of 73 subjects were measured and combined with the secretion rates of non-esterified fatty acids, amino acids, and lactate from adipose tissue, muscle and red blood cells. These data were then used as personalized constraints in GEM iHepatocytes 2322, a model of hepatocytes that extends previous models by including an extensive description of lipid metabolism. This in silico model was used with an untargeted plasma metabolomics approach to identify metabolic changes in response to increased hepatic steatosis. In silico analysis indicated reduced fat oxidation and the depletion of GSH and NAD+ in subjects with high hepatic steatosis, whereas metabolomics analysis revealed decreased plasma levels of GSH metabolism precursors (glycine and glycine precursors: *N*-acetylglycine, betaine, and serine). Considering this result, targeting fat oxidation and GSH and NAD+ availability may provide attractive strategies for NAFLD nutritional treatments. Thus, male C57BL/6J mice were fed a Western diet with or without supplementation with a cocktail containing serine, *N*-acetyl-cysteine (NAC) and a NAD+ precursor (nicotinamide riboside –NR) for 14 days. Supplementation resulted in a 50% reduction in hepatic TAGs and a significant increase in glycine and serine plasma levels. Liver lipidomics analysis revealed that TAGs of short chain lengths, which are preferentially oxidized in the mitochondria, were significantly decreased after supplementation. In addition, supplementation of serine in 6 human patients decreased hepatic steatosis and improved plasma markers of liver function in all subjects. Considering all these results, a cocktail containing L-carnitine, NR, serine, and NAC was proposed to increase fatty acid uptake and oxidation in the mitochondria and to increase GSH availability [136]. 

The above commented findings highlighted the important role of glycine in NAFLD. One of its precursors, betaine (trimethylglycine), is an intermediate in the metabolism of choline. Both glycine and its precursor are an important source of methyl groups in the methionine cycle. Betaine is converted into dimethylglycine as it methylates homocysteine to methionine, which is subsequently used to generate S-adenosylmethionine (SAM), the body’s primary methylating agent. In fact, animal models of NAFLD are commonly induced by choline and methionine-deficient diets [137]. Methyl donor (MeD) supplementation may thus protect against NAFLD. Considering this result, a nutrigenomic approach was applied to investigate the putative preventive role of dietary MeDs in early steatosis resulting from an obesogenic diet [138]. Forty-eight male Wistar rats were randomized into 4 dietary groups: control diet (C), control diet supplemented with methyl donors (C+MeD), high-fat sucrose diet (HFS), and HFS diet supplemented with methyl donors (HFS+MeD). The methyl donor cocktail contained betaine, choline, cobalamin (vitamin B12), and folic acid (vitamin B9). The HFS diet induced liver fat accumulation, which was prevented by MeD supplementation. A transcriptomic and epigenetic approach combining global DNA methylation and methylation changes in specific gene promoters was applied to investigate the mechanisms responsible for these results. A liver mRNA microarray showed significant changes in the genes associated with obesity features between the control and HFS diets (Leptin receptor-Lepr, sterol regulatory element binding transcription factor 2-Srebf2, 1-acylglycerol-3-phosphate *O*-acyltransferase 3-Agpat3, and oestrogen receptor 1-Esr1). Furthermore, although global liver DNA methylation remained unchanged by the HFS diet, it decreased after MeD supplementation in control-fed animals, and methylation profiles from the Srebf2, Agpat3 and Esr1 promoter regions showed changes due to the HFS diet and MeD supplementation [138]. 

The beneficial effects of *n-*3 PUFA on oxidative stress, inflammation, and TAG accumulation in the liver make them attractive candidates for NAFLD treatment. In another recent study, a targeted metabolomics approach was applied to determine the plasma and liver eicosanoid profiles in mice fed a control diet, high-fat diet (HFD) or *n-*3 PUFA supplemented HFD (ω3-HFD) for 4 days [139]. The plasma concentrations of several hydroxyeicosapentaenoic acids (HEPEs) and epoxyeicosatetraenoic acids (EEQs) were reduced by a short-term HFD and were markedly increased by the ω3-HFD, suggesting a critical role of these metabolites in the protective effects of *n-*3 PUFA on NAFLD. Hence, these results provided a solid basis for an effective nutritional intervention with a mixture of HEPEs and EEQs. Mechanistic studies revealed 17,18-EEQ, 5-HEPE, and 9-HEPE as the effective components among these metabolites. Thus, a mixture of these three *n-*3 PUFA metabolites was injected intraperitoneally into mice fed the control diet and HFD. The mixture significantly ameliorated short-term HFD-induced lipid accumulation in the liver and adipose tissue inflammation [139]. 

The application of omics technologies is not only limited to the selection of the best combinations of ingredients for the nutritional intervention. They can be useful to identify which individuals will respond to a particular treatment and, once started, to determine whether a given individual is responding to the treatment without the need of a liver biopsy. This is particularly critical in liver diseases, where no treatment has demonstrated efficacy in more than 50% of patients [117]. Therefore, they can provide a better understanding of this variation in response to nutritional treatments and form the basis of improved, personalized nutrition. In addition, blood, urine, breath, saliva, sweat and faeces constitute non-invasive or minimally invasive biofluids commonly used in omics technologies that could be used as surrogate markers for biopsies to monitor treatment response. For example, we have previously commented that the chronic administration of the multi-ingredient Realsil^®^ ameliorated both liver damage plasma markers and histology. However, this improvement was only seen in approximately 40% of patients with NAFLD and NASH [85]. In a retrospective study, Stiuso et al. applied a lipidomic approach to a selected subset of samples from patients with histologically documented NASH (*n* = 30), both at baseline and 12 months after Realsil^®^ administration [140]. Histology was defined by NAS, with higher scores indicating increased severity. Based on serum oxidative stress markers (thiobarbituric acid reactive substances –TBARS– and nitric oxide) and antioxidant enzyme activities (superoxide dismutase –SOD– and catalase –CAT), patients were divided into 2 groups. The baseline lipidomic profile of both groups of NASH patients showed lower levels of free cholesterol, phosphatidylcholines, and sphingomyelins than those of healthy controls. Supplementation with Realsil^®^ restored the values of these lipid species to normal values only in patient group 2. Interestingly, only this group showed a histologic improvement after Realsil^®^ supplementation, which was measured by the decrease in the NAS score. Thus, the change in the lipidomic profile demonstrated to be a useful biopsy alternative for monitoring nutritional treatment for NASH. 

The results of the “Pioglitazones versus Vitamin E versus Placebo for the Treatment of Non-diabetic Patients with Non-alcoholic Steatohepatitis” (PIVENS) clinical trial constitute another illustrative example to highlight the potential of omics technologies for prediction and monitoring treatment responses, although this study was not based on a multi-ingredient intervention. In this RCT, only 43% of the subjects with NASH receiving vitamin E showed histologic improvement. In addition, 19% of the subjects in the placebo group also demonstrated histologic improvement. Thus, Cheng et al. applied a metabolomics approach to a subset of plasma samples from the PIVENS trial (*n* = 16 vitamin E responders, *n* = 15 vitamin E non-responders, and *n* = 15 placebo responders) to determine whether there were significant differences in the plasma metabolic profiles of subjects who responded to vitamin E at baseline and at the end of treatment compared to those who did not respond [141]. Baseline metabolic profiles differed between vitamin E responders and non-responders. Among the metabolites that were significantly different, phenyl-propionic acid, indole-propionic acid, and asparagine were predictors of the response to vitamin E prior to therapy, whereas the plasma levels of γ-carboxyethylhydroxychroman, γ-palmitoylglycerophosphoethanolamine, and myristoleate were inversely associated with subsequent histologic improvement after vitamin E administration. At the end of treatment, the metabolic profiles between vitamin E responders and non-responders differed in 11 metabolites. From these metabolites, the levels of γ-glutamyl leucine, γ-glutamyl valine, and sphingosine at the end of the treatment were lower in vitamin E responders compared to non-responders. Since their baseline values were comparable in the two groups, lower circulating levels of these metabolites were associated with a histologic response to vitamin E. Interestingly, placebo responders also showed lower levels of γ-glutamyl leucine, γ-glutamyl valine, γ-glutamyl isoleucine, and sphingosine compared to vitamin E non-responders. Lower circulating levels of γ-glutamylated amino acids are indicative of decreased glutathione (GSH) turnover, which reduces the activity of the enzyme GGT. Thus, differences in the metabolic profiles could differentiate those experiencing histologic improvement once in treatment. These findings are in agreement with those of Mardinoglu et al. [136], highlighting the importance of GSH metabolism in NAFLD and the potential of its precursors to be included in multi-ingredient based-therapies. 

All these studies evidence the extraordinary opportunity that omics technologies can offer for designing nutritional interventions for NAFLD. One of the clear advantages is that due to their non-hypothesis-driven nature, they can provide a better understanding of the molecular mechanisms underlying the pathogenesis of NAFLD. However, the advantages are not limited to basic research. Thus, they can be a useful tool for identifying appropriate combinations of ingredients to design effective nutritional interventions. They can also be a useful clinical tool to predict which subjects will or will not respond to nutritional treatment before it starts based on the baseline metabolic profiles, and once started, they can monitor which patients are in fact responding based on changes in metabolic profiles, avoiding invasive liver biopsy. Finally, they can also provide insights about the mechanisms of action of nutritional interventions in NAFLD by revealing the molecular pathways affected by supplementation. Despite all these advantages, omics technologies have been applied only in a limited number of nutritional interventions against NAFLD. Thus, further studies, particularly in humans using a multi-omics approach, are needed to design novel and effective multi-ingredient-based treatments for NAFLD and NASH and to understand their mechanisms of action.

## 5. Conclusions

To sum up, considering all the information reviewed above, it seems evident that adherence to a MedDiet supplemented with certain bioactive compounds and combined with physical exercise may help in the management of liver disease by providing more efficient results than those exerted by current treatments. However, further studies are still required to completely determine the exact components that should be included in these treatments. 

## Figures and Tables

**Table 1:** Human studies aimed to evaluate the effects of MedDiet against NAFLD.

Aim	Design	Dietary Composition	Main Results	Reference
To evaluate the relationship of neglected features of lifestyle with NAFLD and obesity in patients with and without NAFLD.	- 1199 Volunteers (532 with NAFLD and 667 without NAFLD), age 21–60 years. - NAFLD was evaluated by ultrasound (US bright liver score). No liver biopsy during the study. - AMDS assessed based on a 1-week recall computerized questionnaire including physical activity reports. Other lifestyle assessments: western dietary profile score, sun exposure score and a sleep habits questionnaires.	*	- BMI, HOMA-IR and AMDS were revealed as the most potent predictors of liver disease by multiple linear regression analysis although physical activity, western diet and sun exposure were also significant effects. - NAFLD was characterized by increased BMI, HOMA-IR and TAGs, reduced AMDS, sedentary habits, minor sun exposure and higher adherence to western diet.	[40]
To identify associations between the characteristics of patients with NAFLD and the adherence to the MedDiet. Furthermore, the involvement MedDiet in NAFLD was also evaluated.	- 73 adult patients with NAFLD diagnosed by ultrasounds and/or liver histology. - Adherence to the MedDiet estimated with MedDietScore. - Demographic and anthropometric data, body composition analysis and several biochemical and inflammatory markers were determined.	*	- MedDietScore was positively correlated to circulating adiponectin levels while ALT, insulin levels, insulin resistance index and severity of steatosis were negatively correlated. - NASH patients had lower adherence to MedDiet compared to those with simple fatty liver. - One unit increase in the MedDietScore was associated with 36% lower likelihood of having NASH.	[41]
To determine associations between histological characteristics of patients with NAFLD and adherence to the MedDiet.	- Cross sectional study of 82 patients with NAFLD. - The 14-Item Mediterranean Diet Assessment Tool was used to determine the adherence to the MedDiet. - Liver biopsy in all patients.	*	- MedDiet was associated with a reduced probability of having steatohepatitis and steatosis. - HOMA-IR was associated with increased probability of having steatosis and liver fibrosis.	[42]
To evaluate if MedDiet improves insulin sensitivity and reduces steatosis to a greater extent than the currently recommended diet in individuals with NAFLD.	- 12 non-diabetic subjects with biopsy-proven NAFLD volunteers. - Randomized, crossover, 6-week dietary intervention. All the volunteers followed both the MedDiet and a control diet (low fat, high carbohydrate) with a 6-week wash-out period in-between. - Steatosis assessed by magnetic resonance spectroscopy.	- MedDiet: 40% CHO, 40% fat and 20% protein (% of energy). High in MUFAs and *n-*3 PUFAs. Based on a traditional Cretan MedDiet. - Control diet: 50% CHO, 30% fat and 20% protein. Low in saturated and unsaturated fat and high in whole grain foods.	- Insulin sensitivity improved with the MedDiet, while after the control diet remained unchanged. - ↓ Hepatic steatosis after the MedDiet treatment compared with the control diet.	[43]
To determine if weight loss induced by a MedDiet improves liver function in patients with NAFLD.	- 28 obese patients with NAFLD determined by ultrasonography and elevated levels liver enzymes. - Distributed in 2 groups: MedDiet or control group. - Volunteers of MedDiet group attended to 7 intensive counseling sessions for 6 months to increase their adherence to the MedDiet. The patients of the control group only received relevant written recommendations. - The level of adherence to the MedDiet was assessed by MedDietScore.	Not reported.	- Increased MedDietScore and weight loss in volunteers of the MedDiet group. - ↓ ALT (a trend in AST), insulin levels and HOMA-IR only in the MedDiet group. - No similar changes were observed in the control group.	[44]
To evaluate the effectiveness of MedDiet counseling on NAFLD, weight loss, metabolic and liver enzymes.	- 46 patients with NAFLD determined by ultrasound analysis. - Patients received clinical and dietary intervention (based on MedDiet) during 6 months. The counseling was based on monthly meetings (about 45 min each). - The effectiveness was evaluated by liver enzymes, metabolic parameters, cardiovascular risk indexes, NAFLD severity and related indexes.	- MedDiet: 55-60% CHO, 25–30% fat and 10–15% protein (% of energy).	- The number of patients with steatosis grade equal or higher than 2 was reduced from 93% at baseline to 48% at the end of the treatment. Steatosis regressed in 9 patients. - ↓ AST, ALT and GGT during the treatment. - BMI, waist circumference, waist-to-hip ratio, HDL-C, serum glucose, TC/HDL-C, LDL-C/HDL-C, TAGs/HDL-C, AIP, HOMA, FLI, Kotronen index, VAI, NAFLD liver fat score and LAP showed a significant improvement.	[45]
To determine the effectiveness of an increase in the AMDS and the level of physical exercise, evaluating the factors associated with failure.	- 90 adult, obese non-diabetic patients, with NAFLD determined by ultrasound. - 6 months of counseling intervention, based on cognitive-behavioral strategies. - Clinical, laboratory and dietary controls each month.	Not reported.	- ↓ BMI from the first month of intervention. - No significant change of transaminases was observed throughout the study (although levels were already normal at baseline). - ↓ BLS at the end of the study. - AMDS and BMI changes independently explained the variance of decrease of NAFLD.	[46]
To estimate the effect of a Low Glycemic Index MedDiet (LGIMD) on NAFLD.	- Double-Blind RCT composed of 98 patients with moderate or severe NAFLD. - Patients were divided in 2 intervention groups: LGIMD or a control diet during 6 months. - Evaluation of NAFLD score, defined by liver ultrasonography.	- LGIMD: ≤10% saturated fat, high in MUFAs and *n-*3 PUFAs. Only included low glycemic index foods. - Control diet (based on INRAN guidelines): rich in complex CHO and fibre, low in fat and salt.	- Negative interaction between the effect of the LGIMD and time on the NAFLD score - ↓ FLI at six months especially for the LGIMD group. - ↓ TAGs and glycemia were found in both groups after six months. - ↓ GGT and HDL-C were also observed, but only in the LGIMD group.	[47]
To evaluate the therapeutic properties under free living conditions of the Spanish Ketogenic MedDiet (SKMD) in patients with MetS and NAFLD.	- Prospective study using 14 obese men with MetS and NAFLD during 3 months. - Liver disease determined by ALT levels (higher than 40 U/L) and abdominal ultrasonography.	- SKMD: ≤ 30g CHO/day (vegetables), ≥ 30mL virgin olive oil, 200-400 mL wine, unlimited protein (mainly fish). Rich in *n-*3 PUFAs. Included a polyvitamin-mineral supplement.	- ↓ In body weight, LDL-C, ALT, and AST. - ↓ Steatosis degree (complete regression in 21.4% of the patients). - ↓ in all the parameters associated with the MetS: BMI, waist circumference, fasting plasma glucose, TAGs, HDL-C, and blood pressure.	[48]
To analyze the association between adherence to the MedDiet and NAFLD in children and adolescents with obesity.	- 243 young obese patients (age 10–17) with and without liver damage. - NAFLD determined by either abdominal ultrasound or liver biopsy. - Level of adherence to the MedDiet evaluated by the KIDMED.	*	- Low KIDMED score in patients with NASH. - Poor adherence to the MedDiet correlated with liver disease, NAFLD activity score > 5, and grade 2 fibrosis. - Low adherence to the MedDiet correlated with higher values of CRP, fasting insulin and HOMA-IR.	[49]

* Observational study. Abbreviations: AMDS: Adherence to Mediterranean diet score; AIP: Atherogenic index of plasma; ALT: Alanine aminotransferase; AST: aspartate aminotransferase; BLS: Bright Liver Score; BMI: body mass index; CHO: carbohydrates; CRP: C-Reactive Protein; FLI: Fatty Liver Index; GGT: gamma-glutamyltransferase; HDL-C: high density lipoprotein cholesterol; HOMA-IR: homeostasis model assessment–estimated insulin resistance; KIDMED: Mediterranean Diet Quality Index for children and adolescents; LAP: Lipid accumulation product; LDL-C: low density lipoprotein cholesterol; LGIMD: Low Glycemic Index Mediterranean Diet; MedDiet: Mediterranean diet; MetS: metabolic syndrome; MUFAs: monounsaturated fatty acids; NAFLD: nonalcoholic fatty liver disease; NASH: nonalcoholic steatohepatitis; *n-*3 PUFAs: omega-3 polyunsaturated fatty acids; RCT: randomized clinical trial; SKMD: Spanish Ketogenic Mediterranean Diet; TC: total cholesterol; TAGs: triacylglycerides; VAI: visceral adipose index. ↓ means that there is a reduction in the value of the parameter.

**Table 2:** RCT aimed to evaluate the effects of multi-ingredient approaches against NAFLD and NASH.

Ingredients	Design	Main Results	Reference
Silybin (94 mg) + phosphatidylcholine (194 mg) + vitamin E acetate 50% (89.28 mg) (Realsil^®^)	- 138 adult patients with histologically diagnosed NAFLD. HCV-positive patients were included in the trial. - Double-bind, 12 months of supplementation twice daily. - Liver histology during the study.	- ↓ Liver enzyme plasma levels. - ↓ HOMA-IR. - Improvement in liver histology. - BMI normalized in 15% of the patients. - HCV-patients improve fibrogenesis biomarkers.	[85]
Vitamin E + l-gluthatione + l-cysteine + l-methionine + Silybin	- 72 adult patients with NAFLD. - 6 months of treatment twice daily after 3-months of restricted diet. - Ultrasonography to monitor liver damage.	- ↓ Circulating levels of ALT, AST and GGT but AST/ALT unchanged. - ↓ Levels of the steato test. - ↓ The hepatorenal brightness ratio, as an index of hepatic steatosis.	[86]
Vitamin E (30 UI) + (Silybin 125 mg) (Eurosil 85^®^, 210 mg/Tablet; MEDAS SL)	- 36 adult patients with NAFLD based on liver biopsy. - 3 months treatment. - Group I: treated twice daily with the blend and following a hypocaloric diet (1520 kcal, 52% of carbohydrates, 25% of lipids and 23% of proteins) and exercise. Group II: only the hypocaloric diet.	- ↓ Anthropometric parameters in both groups. - ↓ GGT levels in both groups. - ↓ AST and ALT levels only in Group II. - ↓ In both groups of FLI index and NAFLD-FS index. - Only in Group I patients who did not reach a 5% loss of weight also displayed decreased GGT levels, and in the FLI and NAFLD-FS indexes.	[87]
Vitamin E (30 UI) + (Silybin 125 mg) (Eurosil 85^®^, 210 mg/Tablet; MEDAS SL)	- 78 patients with MetS and NAFLD determined by ultrasound. - 3 months of standard MedDiet and exercise (e.g., 15 min walking/day). Group A received 2 tablets daily of Eurosil. Group B only followed the diet and exercise.	- Group A had higher absolute changes from baseline in the biometric parameters (↓ abdominal circumference, BMI, ultrasound measurement of right liver lobe). - Better results of Hepatic Steatosis Index (HSI) and the LAP observed in Group A.	[88]
Vitamin E (300 mg/day) + vitamin C (300 mg/day)	- 23 patients with NASH for 12 months. - BMI measured during therapy. Serum levels of ALT, TRX and hs-CRP measured before and after treatment. - 10 out of the 23 patients underwent liver biopsy before and after treatment.	- BMI unchanged during treatment. - ↓ Serum ALT, TRX and hs-CRP levels after treatment with vitamins. - Improvement in liver biopsies (necro-inflammatory activity in eight cases and fibrosis staging in 4).	[89]
Vitamin E (600 IU/day) + vitamin C (500 mg/day)	- Open-labeled, prospective, randomized study - 57 patients with histologically proven NAFLD and elevated ALT, despite a 3-month reducing diet. - Patients randomized in 2 groups: Group 1 received vitamin E plus vitamin C; Group 2 received ursodeoxycholic acid (UDCA; 10 mg/kg/day). Trial lasted 6 months.	- BMI unchanged after the treatment in both groups. - ↓ ALT and AST in both treatments - Vitamin combination was more effective on ALT levels than UDCA but it was not significant. - ↓ GGT by UDCA but not by vitamins.	[90]
Conjugated Linoleic Acid (CLA; 3000 mg) + vitamin E (400 IU)	- 38 obese NAFLD patients were randomly divided in 2 groups. Intervention group received 3 × 1000 mg softgel of CLA daily with a weight loss diet and 400 IU vitamin E; Control group, received only 400 IU vitamin E weight and the loss diet. Trial extended during eight weeks.	- ↓ BMI, serum oxidative stress, insulin, and improved lipid profile in both groups. - ↓ LDL-C/HDL-C and ALT/AST ratios only in the treated group.	[91]
Docosahexanoic Acid (DHA; 500 mg) + vitamin D (800 IU)	- 41 children and adolescents with NAFLD biopsy-proven followed a 24 weeks treatment. - All patients were included in a lifestyle intervention program: hypocaloric diet (25 ± 30 Kcal/kg/day) and regular physical exercise (twice weekly 1-h physical activity).	- ↓ NAS in the treatment group. - Fibrosis score unchanged but reduction of the activation of HSC and fibrillar collagen content. - ↓ TAG, ALT and HOMA-IR with treatment.	[92]
Pomegranate seed oil (PSO) + brown seaweed extract containing fucoxanthin (Xanthigen)	- Sixteen-week, double-blind, randomized, placebo-controlled study. - 151 non-diabetic obese premenopausal patients (only 41 with NAFLD) - Different doses of PSO and seaweed extract were evaluated in NAFLD and NFL groups.	- The dose of 300 mg PSO + 300 mg brown seaweed extract containing 2.4 mg fucoxanthin ↓ BMI and body and liver fat content in both groups. - ↓ Waist circumference and liver enzymes in NAFLD group. - Weight loss and reduction in body and liver fat content occurred earlier in patients with NFL than in patients with NAFLD.	[93]

Abbreviations: ALT: Alanine aminotransferase; AST: aspartate aminotransferase; BMI: body mass index; CLA: Conjugated Linoleic Acid; CRP: C-Reactive Protein; Docosahexanoic Acid: DHA; FLI: Fatty Liver Index; GGT: gamma-glutamyltransferase; HCV: Hepatitis C virus; HOMA-IR: homeostasis model assessment–estimated insulin resistance; HSC: hepatic stellate cells; LAP: Lipid Accumulation Product; LDL-C: low density lipoprotein colesterol; MetS: metabolic syndrome; NAFLD: nonalcoholic fatty liver disease; NAS: NAFLD Activity Score; NFL: normal liver fat; RCT: Randomized control trial; TAGs: triacylglycerides; TRX: thioredoxin; UDCA: Ursodeoxycholic acid. .↓ means that there is a reduction in the value of the parameter.

**Table 3:** RCT aimed to evaluate the effects of synbiotics against NAFLD and NASH.

Synbiotic	Design	Main Results	Reference
*L. casei*, *L. rhamnosus*, *S. thermophilus*, *B. breve*, *L. acidophilus*, *B. longum*, *L. bulgaricus*, and FOS	- 52 adult overweight and obese patients with NAFLD based on transient elastography and ALT > 60 U/L. - 28 weeks of supplementation (synbiotic or placebo; *n* = 26 per group) twice daily plus lifestyle modification (energy-balanced diet and physical exercise). - No liver biopsy during the study.	- ↓ Fibrosis score (transient elastography) - ↓ Circulating levels of ALT, AST and GGT. - ↓ Blood levels of TNFα and CRP. - ↓ NF-kB p65 in PBMCs. - ↓ HOMA-IR and glucose.	[108]
*L. casei*, *L. rhamnosus*, *S. thermophilus*, *B. breve*, *L. acidophilus*, *B. longum*, *L. bulgaricus*, and FOS	- 42 adult subjects with BMI ≤ 25 and with NAFLD based on transient elastography and ALT > 60 U/L. - 28 weeks of supplementation (synbiotic or placebo; *n* = 21 per group) twice daily plus lifestyle modification (energy-balanced diet and physical exercise). - No liver biopsy during the study.	- ↓ Steatosis and fibrosis (transient elastography). - ↓ Circulating levels of glucose, TAGs, AST and CRP. - ↓ NF-kB p65 in PBMCs.	[109]
*L. casei*, *L. rhamnosus*, *S. thermophilus*, *B. breve*, *L. acidophilus*, *B. longum*, *L. bulgaricus*, and FOS	- 74 adult individuals with NAFLD based on ultrasonography. - 8 weeks of supplementation (synbiotic, *n* = 38 or placebo, *n* = 36) once daily. - No liver biopsy during the study. - No dietary and physical exercise programs.	- ↓ Steatosis severity (ultrasonography) in synbiotic group compared to baseline but not in comparison with the placebo group. - ↓Body weight compared to baseline. - No effects on circulating levels of ALT, AST and CRP.	[110]
*L. casei*, *L. rhamnosus*, *S. thermophilus*, *B. breve*, *L. acidophilus*, *B. longum*, *L. bulgaricus*, and FOS	- 60 adult overweight and obese subjects and with NAFLD based on ultrasonography and ALT > 30 mg/dL - 8 weeks of supplementation (VitE, synbiotic, symbiotic + VitE or placebo; *n* = 15 per group) twice daily. - No liver biopsy or ultrasonography during the study. - No dietary and physical exercise programs.	- ↓ Circulating levels of ALT, AST, ALP. - ↓ Blood levels of TNFα, leptin. - ↓ Circulating levels of TAGs, TC, LDL-C, APOB100/APOA1. - ↓ Circulating levels of glucose, insulin. - Synbiotic +Vit E produced a greater drop of the circulating levels of LDL-C, TNFα, ALT, AST, ALP, leptin, insulin and glucose than the symbiotic.	[111,112]
*B. longum* and FOS	- 66 adult patients with biopsy-proven NASH, abnormal serum transferase levels and steatosis based on ultrasonography. - 4 initial weeks of lifestyle modification (energy-balanced diet and physical exercise). - 24 weeks of supplementation (synbiotic, *n* = 34 or placebo, *n* = 32) twice daily plus lifestyle modification. - Liver biopsy also performed at the end of the study.	- ↓ Steatosis severity and NASH activity index (liver biopsy). - ↓ Circulating levels of AST, TNFα, CRP and LDL-C. - ↓ HOMA-IR. - ↓ Serum endotoxin.	[113]
*L. reuteri* plus inulin and guar gum	- 50 adult patients with MetS and biopsy-proven NASH. - 3 months of supplementation (synbiotic, *n* = 27 or placebo, *n* = 23) twice daily plus lifestyle modification (hypocaloric diet). - No physical exercise program. - No liver biopsy during the study.	- ↓ Steatosis severity (PDFF) in synbiotic group compared to baseline but not in comparison with the placebo group. - ↓Body weight, waist circumference and BMI compared to baseline. - ↑ Circulating levels of LPS (in both placebo and synbiotic groups). - ↓ Serum levels of uric acid - No effects on circulating levels of ALT, AST, ALP, GGT, glucose and lipids.	[114]

Abbreviations: ALT: Alanine aminotransferase; ALP: alkaline phosphatase; APOA1: Apolipoprotein A1; APOB100: apolipoprotein B100; AST: aspartate aminotransferase; BMI: body mass index; CRP: C-Reactive Protein; FOS: fructooligosaccharide; GGT: gamma-glutamyltransferase; HOMA-IR: homeostasis model assessment–estimated insulin resistance; LDL-C: low density lipoprotein colesterol; LPS: lipopolysaccharides; MetS: metabolic syndrome; NAFLD: nonalcoholic fatty liver disease; NASH: nonalcoholic steatohepatitis; NF-kB p65: p65 subunit of Nuclear factor k-B; PBMCs: peripheral mononuclear cells; PDFF: proton density fat fraction; RCT: randomized clinical trial; TC: total cholesterol; TNF-α: tumor necrosis factor-α; TAGs: triacylglycerides. ↓ means that there is a reduction in the value of the parameter.

## Abbreviations

1-acylglycerol-3-phosphate *O*-acyltransferase 3 (Agpat3), Adherence to Mediterranean diet score (AMDS), alkaline phosphatase (ALP), alanine aminotransferase (ALT), AMP-activated protein kinase (AMPK), aspartate aminotransferase (AST), body mass index (BMI), conjugated linoleic acid (CLA), cardiovascular disease (CVD), catalase (CAT), C-Reactive Protein (CRP), docosahexaenoic acid (DHA), epoxyeicosatetraenoic acids (EEQs), fatty liver index (FLI), fibrosis score (FS), fructooligosaccharide (FOS), gamma-glutamyltransferase (GGT), genome-wide associations studies (GWAS), glutathione (GSH), high density lipoprotein cholesterol (HDL-C), high-fat diet (HFD), high-fat sucrose diet (HFS), homeostasis model assessment–estimated insulin resistance (HOMA-IR), hydroxyeicosapentaenoic acids (HEPEs), intestinal microbiota (IM), leptin receptor (Lepr), low density lipoprotein cholesterol (LDL-C), lipopolysaccharides (LPS), Mediterranean diet (MedDiet), metabolic syndrome (MetS), methyl donor (MeD), monounsaturated fatty acids (MUFAs), *n-*3 PUFA supplemented HFD (ω3-HFD), non-alcoholic fatty liver disease (NAFLD); NAFLD activity score (NAS), non-alcoholic steatohepatitis (NASH), nicotinamide ribotide (NR), nuclear magnetic resonance (NMR), estrogen receptor 1 (Esr1), patatin-like phospholipase domain containing 3 (PNPLA3), p65 subunit of Nuclear factor k-B (NF-kB p65), peripheral mononuclear cells (PBMCs), proton density fat fraction (PDFF), peroxisome proliferator activated receptor (PPAR), polyunsaturated fatty acids (PUFAs), randomized clinical trial (RCT), S-adenosylmethionine (SAM), short chain fatty acids (SCFAs), superoxide dismutase (SOD), sterol regulatory element binding transcription factor 2 (Srebf2), thiobarbituric acid reactive substances (TBARS), toll-like receptors (TLR), transmembrane 6 superfamily member 2 (TM6SF2), tumor necrosis factor-α (TNF-α), triacylglycerides (TAGs), type 2 diabetes (T2D), very-low density lipoproteins (VLDL), World Health Organization (WHO).
